# Editorial: Restoring endoplasmic reticulum proteostasis to treat neurological disorders

**DOI:** 10.3389/fphar.2023.1176805

**Published:** 2023-03-09

**Authors:** Danilo B. Medinas, Najla Arshad, Sonam Parakh, Sayuri Miyamoto, Vanessa Olzon Zambelli

**Affiliations:** ^1^ Department of Biochemistry, Institute of Chemistry, University of São Paulo, São Paulo, SP, Brazil; ^2^ Biomedical Neuroscience Institute, Faculty of Medicine, University of Chile, Santiago, Chile; ^3^ Center for Geroscience, Brain Health, and Metabolism, Santiago, Chile; ^4^ Program of Cellular and Molecular Biology, Center for Molecular Studies of the Cell, Institute of Biomedical Sciences, University of Chile, Santiago, Chile; ^5^ Department of Immunobiology, Yale University School of Medicine, New Haven, CT, United States; ^6^ Department of Biomedical Sciences, Centre for MND Research, Faculty of Medicine, Health, and Human Sciences, Macquarie University, Sydney, NSW, Australia; ^7^ Laboratory of Pain and Signaling, Butantan Institute, Sāo Paulo, SP, Brazil

**Keywords:** neurological disorders, ischemia-reperfusion injury, spinal cord injury, endoplasmic reticulum, proteostasis, unfolded protein response, small molecules, drug discovery

A wide range of pathological conditions can affect normal functioning of the nervous system, constituting the second leading cause of death and the major factor for disability worldwide (Carroll, 2019). The etiology of neurological disorders is varied and complex, including genetic mutations, trauma, ischemia-reperfusion (IR) injury, aging, and many others. Most of these pathologies are incurable, meaning there is an urgent need for novel therapeutic strategies to sustain neuronal function and prevent cell death. Perturbations to proteostasis (i.e., protein homeostasis) of the nervous system have emerged as important pathogenic mechanisms contributing to protein aggregation, synaptic failure, neuroinflammation, among other disease hallmarks (Hetz, 2021). The endoplasmic reticulum (ER) is a major organelle involved in protein biogenesis, integrating many proteostasis pathways critical to cell migration and differentiation during neurodevelopment, in addition to maintenance and remodeling of the intricate neuronal morphology and synaptic connections over the organism’s lifespan (Godin et al., 2016; Martínez et al., 2017; Martínez et al., 2018). In this Research Topic, we have gathered original and review manuscripts illustrating the potential of manipulating ER proteostasis to tackle neurological disorders.

The interplay between proteostasis regulation and myriad cellular functions offers multiple points of intervention for therapeutic exploration. Studies in patients and experimental models established that ER proteostasis is compromised across several conditions, including Alzheimer’s disease (AD), Parkinson’s disease (PD), Huntington’s disease (HD), amyotrophic lateral sclerosis (ALS), stroke, among others (Hetz and Saxena, 2017). The proteostasis network in the ER has many components such as chaperones and folding catalysts, proteasomal and lysosomal degradation systems, and the unfolded protein response (UPR) that are disrupted in neurological disorders (Balch et al., 2008; Labbadia and Morimoto, 2015). Moreover, ER proteostasis also relies on the structural and functional integration of the ER with other organelles such as the mitochondria, in addition to being modulated by lipid metabolism (Wu et al., 2018). The growing mechanistic understanding of how these cellular systems are involved in the etiology of neurological conditions can provide the conceptual framework for the design of multimodal pharmacological interventions.

In the review manuscript by Pérez-Arancibia et al., the authors provide a detailed discussion on the use of small molecules to treat neurological disorders, focusing mainly on PD and HD, the most common movement disorder caused by a dysfunction in the dopaminergic circuits. Molecules that have entered clinical trials are highlighted, which can be classified as synthetic or natural compounds. In addition, some emphasis is placed on drug repurposing and selected examples under preclinical testing, where the authors present mechanism of action of different compounds and critically discuss the challenges for their development into novel drugs. A common pathogenic event in neurodegenerative diseases like PD and HD is protein misfolding and aggregation, which can impact ER proteostasis at multiple levels (Hetz and Saxena, 2017). The studies discussed by Pérez-Arancibia et al. clearly show that inhibiting the accumulation of misfolded and aggregated proteins holds great therapeutical promise by rescuing multiple cellular pathways intimately linked to ER proteostasis.

A special feature of the nervous system is the structure afforded by the tight arrangement of endothelial cells of the vasculature along with glial cells and neurons, a system known as the blood brain barrier (BBB) or blood spinal cord barrier (BSCB) that shields against potentially toxic substances in the bloodstream. The BBB and BSCB also regulates the supply of nutrients and modulate the crosstalk between the nervous and immune systems, guaranteeing tissue homeostasis. The breakdown of the neurovascular unit has been associated with numerous neurodegenerative diseases and other pathological conditions marked by inflammatory response (Persidsky et al., 2006). In this context, Deng et al. discuss the impairment of the BSCB under traumatic spinal cord injury (t-SCI), providing a meta-analysis of treatments that rescue BSCB in rat models of t-SCI. Excitingly, the authors have found that ER stress is a major contributor to BSCB damage, offering novel therapeutic targets. A common outcome of the experimental treatments examined by the authors is the upregulation of tight junction (TJ) and adhesion junction (AJ) proteins, which are an integral part of the BSCB, promoting close contacts between endothelial cells and regulating the nervous system permeability. Considering that TJ and AJ are constituted of membrane proteins produced in the ER, ER stress mitigation can lead to increased levels of TJ and AJ proteins and improved BSCB integrity.

IR injury is another pathogenic insult that involves ER stress followed by neuronal demise. The original research contributed by Yuan et al. describes the protective effects of AA147, a pharmacologic agonist of the activating transcription factor 6 (ATF6). ATF6 is a sensor and signal transducer of the UPR, which responds to ER stress by translocating to the nucleus to promote adaptative gene expression programs to restore ER proteostasis. The authors show that AA147 treatment improved the neurological score of mice subjected to cardiac arrest followed by cardiopulmonary resuscitation. The beneficial effects of AA147 were accompanied by reduced apoptosis of neurons and diminished oxidative damage, in addition to increased levels of ER chaperones and antioxidant enzymes. Besides activating ATF6, AA147 also upregulates nuclear factor E2-related factor 2 (Nrf2), a major transcription factor inducing the antioxidant response. Thus, Yuan et al. provide compelling evidence for the therapeutic value of a single treatment targeting both ER stress and oxidative stress. Although these pathogenic mechanisms may be understood as separate cellular events, they are closely related and may act synergistically in myriad diseases. Strategies that interrupt feed forward pathogenic cascades such as ER stress and oxidative stress hold great translational promise.

The original contribution by Bhat et al. provides an elegant approach to tackle pathogenic protein-protein interaction occurring under ischemic insult. The authors identified that the UPR-induced transcription factor C/EBP homologous protein (CHOP), which mediates apoptotic cascades under irremediable ER stress, interacts with the gamma-aminobutyric acid (GABA) receptor, preventing its trafficking to the cell surface. Consequently, a loss of inhibitory modulation by GABA and neuronal death by excitotoxicity is reported. Through a peptide-based approach, Bhat et al. showed that blocking CHOP interaction with GABA receptor in cultured neurons under pathogenic conditions normalizes neuronal activity and rescues cellular viability. Further studies investigating this approach *in vivo* are eagerly awaited.

Overall, this Research Topic of reports illustrates the value of targeting ER proteostasis to treat neurological conditions, where basic and translational research should advance in parallel to identify and explore therapeutic targets through a discovery pipeline supported by investigation of pathogenic mechanisms in pre-clinical models coupled to drug screenings of effective disease modifiers.
